# TNF in the liver: targeting a central player in inflammation

**DOI:** 10.1007/s00281-022-00910-2

**Published:** 2022-02-04

**Authors:** Gisa Tiegs, Andrea K. Horst

**Affiliations:** 1 Institute of Experimental Immunology and Hepatology, University Medical Center Hamburg-Eppendorf, Hamburg, Germany; 2 Hamburg Center for Translational Immunology, University Medical Center Hamburg-Eppendorf, Hamburg, Germany

**Keywords:** TNF signalling, Inflammation, Apoptosis, Viral hepatitis, Non-alcoholic fatty liver disease, Autoimmune hepatitis, Primary sclerosing cholangitis

## Abstract

Tumour necrosis factor-α (TNF) is a multifunctional cytokine. First recognized as an endogenous soluble factor that induces necrosis of solid tumours, TNF became increasingly important as pro-inflammatory cytokine being involved in the immunopathogenesis of several autoimmune diseases. In the liver, TNF induces numerous biological responses such as hepatocyte apoptosis and necroptosis, liver inflammation and regeneration, and autoimmunity, but also progression to hepatocellular carcinoma. Considering these multiple functions of TNF in the liver, we propose anti-TNF therapies that specifically target TNF signalling at the level of its specific receptors.

## Introduction

TNF is a pro-inflammatory cytokine critically involved in autoimmune diseases. Therefore, TNF inhibitory approaches successfully entered the clinic for therapy of e.g. rheumatoid arthritis (RA), psoriasis, and inflammatory bowel disease (IBD) [1]. Unfortunately, not all patients respond to anti-TNF therapy. On the contrary, paradoxically and in rare cases, anti-TNF therapy can trigger autoimmune-like diseases such as a lupus-like syndrome or autoimmune hepatitis (AIH) [2] and might induce drug-induced liver injury (DILI) [3]. These clinical observations are probably due to the pleiotropic molecular properties of TNF. TNF induces inflammation, apoptosis, and necroptosis, but also cell survival and proliferation primarily via activation of its ubiquitously expressed TNF receptor 1 (TNFR1) [2].

In the liver, TNF mediates hepatocellular death and seems to be involved in sterile inflammation, viral hepatitis, AIH, and hepatocellular carcinoma (HCC) [4–7]. The multifunctional properties of TNF in the liver apparently result from activation of a multitude of downstream signals resulting in functionally different, sometimes contrary cellular responses such as cell death and inflammation on the one hand, and cell survival on the other hand. These properties of TNF make it necessary to study the exact molecular function of TNF and its receptors in a specific hepatic disease to identify the most relevant target for therapeutic intervention.

## TNF signalling

**Fig. 1:**
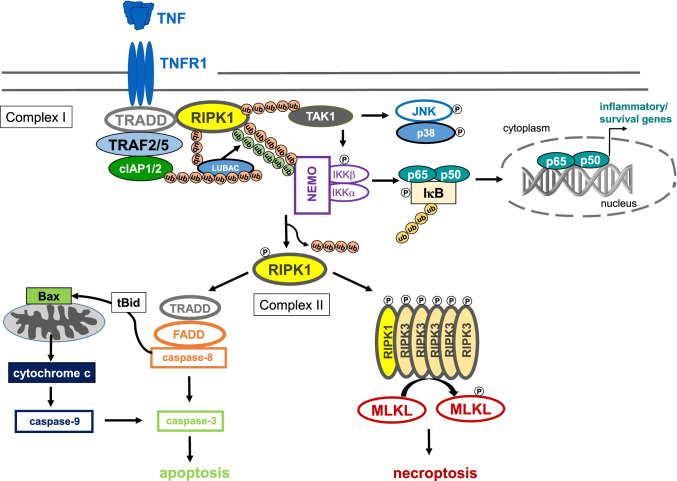
TNFR1 signalling. Binding of TNF to TNFR1 results in the formation of complex I which consists of the membrane-bound adaptor molecules TNFR1-TRADD-RIPK1-TRAF2 or TRAF5 that associate with the E3 ubiquitin ligases cIAP1 and cIAP2, which add K63-linked polyubiquitin chains to RIPK1 and other components of the signalling complex. This results in recruitment of linear ubiquitin chain assembly complex (LUBAC), which adds M1-linked linear polyubiquitin chains to RIPK1. Subsequently, via activation of TAK1, the Jun/p38 signalling pathways as well as the IKK complex, consisting of IKKα, IKKβ, and the regulatory subunit NEMO, are activated. IKK phosphorylates the inhibitor of NFκB, IκB, which is then degraded following K48 polyubiquitination, giving rise to activated NFκB and its translocation into the nucleus. By binding to specific promoter regions of cytokine and chemokine genes, but also for genes of anti-apoptotic, cytoprotective proteins (see text for details), NFκB mediates either inflammation or cell survival. Upon recruitment of FADD to TRADD, the death-inducing signalling complex (complex II) is formed, which in turn recruits pro-caspase 8 to finally execute the proapoptotic downstream pathway of TNFR1. In hepatocytes, TNF-induced apoptosis requires the activation of the mitochondrial pathway that involves cleavage of BH3 interacting-domain death agonist (Bid) to truncated Bid (tBid), activation of Bax, formation of mitochondrial permeability transition pores, release of cytochrome c from the mitochondria, and finally activation of caspase 9 and caspase 3. Alternatively, in the presence of phosphorylated RIPK1, RIPK3 can be recruited into the complex which in turn phosphorylates MLKL to induce necroptosis. Necroptotic cell death is caspase-independent and represents a pro-inflammatory form of cell death

**Fig. 2:**
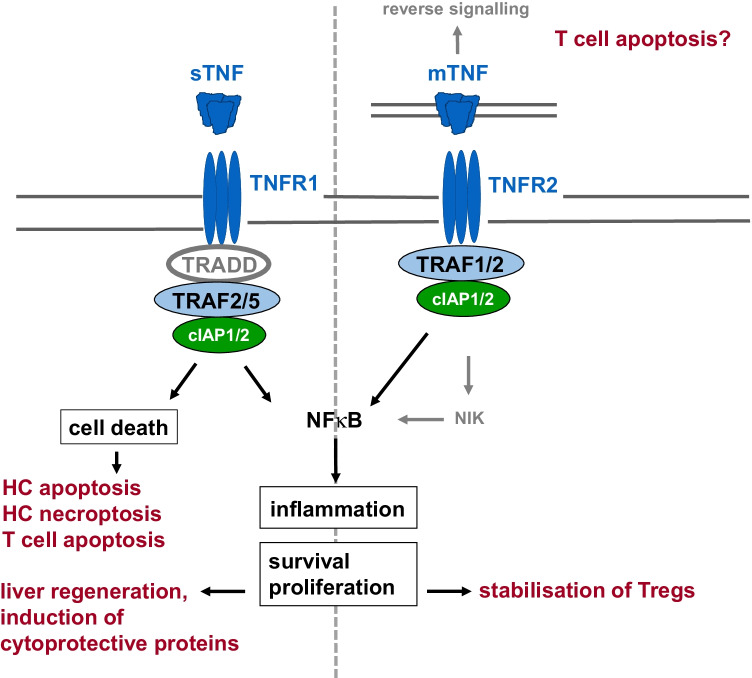
Overview of the different functions of TNFR1 and TNFR2. TNFR1 is activated by sTNF and mTNF whereas TNFR2 is preferentially activated by mTNF. TNFR1 contains a death domain and can therefore execute cell death signals. TNFR1 signalling that leads to the activation of NFκB (cf. Fig. 1) mediates inflammation as well as cell survival and proliferation. TNFR2 does not contain a death domain. Recruitment of TRAF1 and 2 as well as cIAP1 and 2 to the intracellular domain of TNFR2 results in canonical and, via activation of NFκB-inducing kinase (NIK), to non-canonical NFκB activation. Besides induction of inflammation, cell survival, and proliferation, TNFR2 was supposed to cooperate with TNFR1 to induce cell death by down-modulation of TRAF2 and cIAPs, thereby inhibiting the anti-apoptotic NFκB signalling pathway (not shown, see text). Moreover, it has been assumed that mTNF might function as receptor for an activating antibody, thereby inducing apoptosis by reverse signalling (see text)

TNF is produced as a 26-kDa transmembrane precursor protein (mTNF) that is proteolytically cleaved by TNF-converting enzyme (TACE, ADAM17) to generate a soluble 17-kDa protein (sTNF). Both sTNF and mTNF are bioactive and signal as homotrimers via two structurally distinct membrane receptors, p55 TNFR1 and p75 TNFR2, which are trimerized upon ligand binding [8]. While TNFR1 is ubiquitously expressed, TNFR2 expression is essentially restricted to haematopoietic and endothelial cells during homeostasis and can be further induced in response to inflammation. mTNF, as well as sTNF, can stimulate TNFR1, while TNFR2 is preferentially activated by mTNF. Binding of TNF to TNFR1, which contains a death domain, results in the formation of complex I which consists of the membrane-bound signalling core TNFR1—TNFR1-associated death domain (TRADD)—receptor-interacting protein kinase (RIPK) 1—TNF receptor–associated factor (TRAF) 2 (Fig. 1). TRAF2 binds to the cellular inhibitors of apoptosis proteins 1 (cIAP1) and 2 (cIAP2). Polyubiquitin chains added to this complex assure appropriate protein scaffolding and downstream signal activation. Formation of complex I results in the activation of TGFβ-activated kinase 1 (TAK1) which further activates c-Jun N-terminal kinase (JNK)/p38 signalling as well as the inhibitor of κB (IkB) kinase (IKK). The IKK complex consists of three subunits, IKKα, IKKβ, and the regulatory subunit nuclear factor κ B (NFκB) essential modulator (NEMO, also known as IKKγ). Once activated, IKK phosphorylates the NFκB inhibitor-α (IκBα) which is degraded upon K48-linked polyubiquitinylation, finally allowing the transcription factor NFκB to translocate into the nucleus and to activate transcription of numerous of its target genes involved in inflammation, cell survival, and proliferation [2, 8]. Homozygote mice lacking the p65/RelA component of NFκB exhibit embryonic lethality due to massive liver degeneration [9]. Moreover, NFκB mediates liver regeneration that requires activation of TNFR1 but not TNFR2 (cf. Fig. 2) [10, 11]. The cytoprotective effects of TNF are mediated by NFκB-inducible anti-apoptotic proteins such as cIAPs, cellular FLICE-like inhibitory protein (c-Flip), A20, X-chromosome linked inhibitor of apoptosis protein (XIAP), and B cell lymphoma-extra large (Bcl-X_L_), most of them interacting with signalling molecules of the death signalling pathway [12].

The death-inducing signalling complex (complex II) is formed upon recruitment of Fas-associated death domain (FADD) to TRADD, which in turn recruits pro-caspase 8 to finally execute the proapoptotic downstream pathway of TNFR1 [2, 8]. Activated caspase 8 proteolytically cleaves pro-caspase 3 to activate the executer caspase 3, which could be sufficient to mediate apoptosis. In hepatocytes, TNF-induced activation of caspase 3 requires additional signals of a mitochondrial pathway that involves activation of Bcl-2-associated X protein (Bax), a proapoptotic member of the B cell lymphoma 2 (Bcl-2) family, formation of mitochondrial permeability transition (MPT) pores, and release of cytochrome c [5, 13]. Alternatively, in the presence of phosphorylated RIPK1, RIPK3 can be recruited into the complex which in turn phosphorylates mixed lineage kinase domain-like protein (MLKL) to induce necroptosis. Necroptotic cell death is caspase-independent and represents a pro-inflammatory form of cell death (Fig. 1) [2, 8]. TNFR2 does not contain a death domain and therefore cannot induce cell death. However, via recruitment of TRAFs, TNFR2 activates NFκB and thereby induces inflammation and cell survival [2, 8]. Notably, substantial evidence points to a role for TNFR2 in regulatory T cell (Treg) expansion, immunosuppressive function, and stability (Fig. 2) [14, 15].

## TNF mediates hepatocellular death

In 1975, Carswell et al. [16] characterized the serum factor that has been described to mediate necrosis of solid tumours caused by bacterial toxins, i.e. endotoxin or lipopolysaccharides (LPS), and named it TNF. TNF was toxic for neoplastic cell lines but not for mouse embryonic cells. Later, it was shown that d-galactosamine (d-GalN), a transcriptional inhibitor, sensitizes wild-type mice to lethal toxicity of LPS and TNF [17] and that administration of either LPS or TNF to d-GalN-sensitized mice caused fulminant hepatitis [18]. d-GalN is primarily metabolized in hepatocytes where it depletes uridine nucleotides and thereby functions as transcriptional inhibitor [19]. Transcriptional inhibition sensitised hepatocytes towards induction of apoptosis in response to TNF [20], an effect that is mediated by TNFR1 (Figs. 1 and 2) [21, 22]. Pretreatment of mice with TNF alone, without transcriptional inhibition, protected them from apoptotic liver damage subsequently induced by D-GalN/TNF administration, an effect that was mediated by TNF-inducible NFκB-dependent cytoprotective proteins such as A20, which inhibited TNF-induced mitochondrial apoptosis in the mouse liver by down-modulation of Bax [23, 24]. Moreover, interference with linear polyubiquitination of RIPK1 in complex I that is necessary for TNFR1-mediated NFκB activation sensitised hepatocytes to TNF-induced apoptosis even in the absence of transcriptional inhibition and exacerbated TNF-mediated liver damage in mice [25]. In addition, the protective effect of TNF administration was most likely mediated by TNFR1, since nuclear translocation of NFκB was almost completely inhibited in *Tnfr1*^*−/−*^ mice but fully inducible in *Tnfr2*^*−/−*^ mice following combined activation of both TNF receptors in the murine liver (cf. Fig. 2) [26, 27].

Besides induction of hepatocellular apoptosis, TNF induces necroptosis in the mouse liver in models of inflammatory liver injury (Figs. 1 and 2). In the mouse model of concanavalin A (ConA)-induced hepatitis, which depends on activation of CD4^+^ T cells and NKT cells [28, 29], TNF mediates liver damage independently of transcriptional inhibition [30]. Notably, in ConA-hepatitis, TNF mediates liver injury in its soluble and transmembrane-bound precursor form that depends on activation of both TNF receptors [27].

In this model, TNF induces caspase-8- and caspase-3-independent liver cell death [31–33] which is probably mediated by prolonged JNK activation [32, 34]. Notably, the JNK pathway has been implicated in both hepatocyte proliferation and apoptosis (reviewed in [35]). Liver cell death in the ConA-model resembles necroptosis, as the disease-associated molecular pattern (DAMP) IL-33, which is associated with necroptotic cell death, is released from hepatocytes upon ConA treatment [36, 37]. Moreover, inhibitors of necroptosis protected mice from ConA-induced liver injury [38].

 Until now, a role of TNFR2 for liver injury was scarcely described. In 1998, Douni and Kollias [39] reported that mice transgenic for the human TNFR2 developed a severe inflammatory syndrome, including inflammatory liver disease even in the absence of endogenous TNFR1, which supported the notion that TNFR2 primarily drives inflammation by increased NFκB activation. However, in a model of immune-mediated liver injury that depends on both TNF receptors, we provided evidence for cooperative cell death signalling of TNFR1 and TNFR2 by showing that bone marrow chimeric mice that express TNFR2 in leucocytes but not in parenchymal cells were protected from liver damage [40]. Mechanistically, cooperative cell death signalling of both TNFRs was explained by TNFR2-induced down-modulation of TRAF2 and cIAPs, thereby affecting the TNFR1-dependent anti-apoptotic NFκB signalling pathway [41].

## Contribution of TNF to sterile liver inflammation

### Acetaminophen

The prominent role of TNF for induction of liver cell death prompted many investigators to study the role of TNF and its receptors in liver disease, liver regeneration, and HCC in mouse models and in patients’ studies (summary of major TNF effects in Table 1). The term sterile liver inflammation was established by showing that the NLR family pyrin domain containing 3 (NLRP3) inflammasome contributed to liver damage without infection, e.g. inducible by the anti-pyretic and analgesic drug acetaminophen (APAP, *N*-acetyl-4-aminophenol; reviewed in [42]). APAP is one of the most commonly used over-the-counter drugs taken worldwide. APAP induces acute liver failure upon suicidal intent or unintentional overdose [43]. Sterile inflammation in the liver is triggered in response to hepatocyte death probably induced by RIPK3-mediated necroptosis [44], which results in the release of DAMPS such as high-mobility group box 1 protein (HMGB1), heat shock proteins (HSPs), and DNA fragments (reviewed in [45]). DAMPS activate toll-like receptors (TLRs) expressed by monocytes/macrophages and activate a pro-inflammatory response via release of cytokines and chemokines [45]. Although, despite production of pro-inflammatory cytokines such as IL-1β, IL-6, and TNF, TNF-deficient mice were not protected from APAP liver toxicity [46] and mice lacking TNFR1 even developed aggravated liver damage [47]. These findings point to a beneficial role of TNF most likely initiating tissue repair in response to APAP-induced liver damage [45].

**Table 1:** Major effects of TNF on liver pathology

Liver disease	Major TNF effect
*APAP-induced liver damage*	Tissue repair [45]
*Alcoholic liver disease (ALD)*	None [52]
*Non-alcoholic fatty liver disease (NAFLD) and non-alcoholic steatohepatitis (NASH)*	Increased serum levels and hepatic expression of TNF and TNFR1, correlating with disease activity in NASH patients [64–66], TNFR1-dependent attenuation of insulin resistance and liver injury in a mouse model [68], Normalisation of liver biochemistry in a NASH patient treated with adalimumab for co-existing RA [69]
*Viral hepatitis*	
HBV	Induction of cell death in HBV-infected hepatocytes, NFκB-dependent blockade of HBV replication [75, 83, 85]
HCV	Induction of apoptosis in HCV-infected Huh-7.5 cells [77]
*Autoimmune liver disease*	
AIH	*TNF* polymorphism associated with type 1 AIH susceptibility [93], Successful treatment of AIH with infliximab [6]
PBC	Pathway analysis provided evidence for enhanced TNF signalling in PBC pathogenesis [87], Stabilisation of liver function in PBC patients treated with TNF antagonists for co-existing RA [90, 91]
PSC	High expression levels of *TNF* in innate-like CD4^+^ T cells from PSC patients [113], Moderate effectiveness of anti-TNF therapies in PSC/IBD patients [114, 115]

*AIH*, autoimmune hepatitis; *APAP*, acetaminophen; *HBV*, hepatitis B virus; *HCV*, hepatitis C virus; *IBD*, inflammatory bowel disease; *PBC*, primary biliary cholangitis; *PSC*, primary sclerosing cholangitis; *RA*, rheumatoid arthritis; *TNF*, tumour necrosis factor; *TNFR*, TNF receptor

### Alcoholic liver disease (ALD)

ALD is one of the most common causes of chronic liver disease worldwide that may progress from alcoholic steatohepatitis to fibrosis, cirrhosis, and HCC [48]. Metabolic products of alcohol could directly cause liver cell death. Moreover, epithelial barrier disruption in the gut due to intestinal microbial dysbiosis, bacterial translocation, and increased LPS concentrations in the portal blood are considered to be responsible for activation of the innate immune response in the liver, which is associated with an increased production of pro-inflammatory cytokines such as TNF [48]. Indeed, RIPK3-dependent necroptosis was shown to be responsible for murine ALD [49]. Moreover, results from animal studies using either TNF neutralizing antibodies or TNFR1-deficient mice provided evidence for protection from chronic alcohol exposure [50, 51]. However, a multicentre clinical trial using the dimeric TNF-binding fusion protein etanercept as a treatment of patients with moderate to severe alcohol hepatitis failed because of serious adverse events such as increased infection rates and a significantly higher mortality rate after 6 months [52], clearly indicating that TNF blockade is not suitable for treatment of patients with ALD.

### Non-alcoholic fatty liver disease (NAFLD)

Due to the growing incidence of obesity worldwide, NAFLD is a major cause of chronic liver disease with increasing prevalence. NAFLD is characterized by hepatic steatosis which may progress to non-alcoholic steatohepatitis (NASH) and development of fibrosis, cirrhosis, and HCC [53]. Co-morbidities are frequent and comprise the metabolic syndrome, insulin resistance, hypertension, and increased cardiovascular risk [53]. Multiple metabolic pathways are affected during NAFLD including metabolism of glucose, lipids, amino acids, and bile acids (BA). Within the liver, metabolic deterioration results in lipotoxicity, mitochondrial dysfunction, and endoplasmatic reticulum (ER) stress that induce apoptosis, necroptosis, and probably other types of lytic cell death in hepatocytes [54]. In response to cell lysis, released DAMPS activate their receptors expressed by macrophages and/or hepatocytes and induce inflammation. HMGB1 activates TLR4, DNA fragments TLR9, and ATP, the NLRP3-inflammasome amplifying receptor P2X7 [42, 55]. In addition, NAFLD/NASH is associated with intestinal dysbiosis, probably resulting from intake of an inconvenient diet, which leads to a dysfunction of the epithelial barrier in the gut, associated with increased permeability and translocation of bacteria and bacterial products into the portal blood. Bacterial products such as LPS that function as pathogen-associated molecular patterns (PAMPs) also activate TLRs, thereby amplifying the inflammatory response in the liver, characterized by increased production of cytokines such as TNF, IL-1, and IL-17A as well as the chemokines CCL2 and CCL5 that further recruit inflammatory monocytes into the liver [53, 56, 57]. Moreover, changes in BA metabolism, associated with enhanced systemic levels of potentially cytotoxic BA, have been identified in NASH patients (reviewed in [58]). Altered *ratios* of primary to secondary BA levels underscore the impact of microbial alterations in the gut of NASH patients [59]. In addition, expression of enzymes for BA synthesis and BA transporters has been shown to be regulated by cytokines such as TNF and interferon-γ [60]. Interestingly, BA receptors such as the nuclear farnesoid X receptor (FXR), the Takeda G protein–coupled receptor 5 (TGR5 also known as G protein–coupled BA receptor 1 (GPBAR1)), and the vitamin D receptor (VDR) have anti-inflammatory and immune-regulatory properties by shaping the innate and the adaptive immune system (reviewed in Evangelakos et al. [58]). Therefore, numerous FXR agonist such as obeticholic acid entered clinical trials for therapy of NASH [53, 56, 58, 61].

TNF is a mediator of insulin resistance in obesity, where it is overexpressed in adipose tissue and induces the release of the pro-inflammatory lipokine leptin from adipocytes [62, 63]. In NAFLD/NASH patients, elevated serum concentrations of TNF and soluble TNFR1 have been determined, which were associated with disease severity [64, 65]. Moreover, increased expressions of TNF in the liver and adipose tissue as well as enhanced hepatic TNFR1 transcripts were detected in NASH patients, again correlating with disease progression [66]. In a NASH mouse model, where wild-type and TNFR1/TNFR2-double knockout mice were fed a methionine and choline-deficient diet, Kupffer cell activation and expression of pro-inflammatory molecules were significantly suppressed upon TNFR deficiency. Accordingly, hepatic stellate cell activation, liver steatosis, and fibrosis were attenuated in these animals [67]. In a recent report, Wandrer et al. [68] administered a high-fat diet to humanized TNFR1 knock-in and control mice for 24 weeks before they started a therapeutic intervention with an anti-human TNFR1 antibody for another 8 weeks. This curative treatment, specifically blocking TNFR1, significantly reduced the activation of the mitogen-activated protein kinase (MAPK) pathway in the liver, insulin resistance, liver injury, steatosis, and fibrosis [68]. Hence, blockade of TNFR1 might represent a promising approach for treatment of NASH. In a case report, Schramm and co-workers reported a patient with NASH who developed rapid normalisation of liver biochemistry during treatment of an associated RA with the humanized anti-TNF antibody adalimumab [69]. However, up to now, clinical trials targeting TNF or its receptors as a therapy for NAFLD/NASH are not available.

## Role of TNF in viral hepatitis

Infection with hepatitis B virus (HBV) and hepatitis C virus (HCV) elicits an antiviral immune response characterized by an innate type I interferon response and an innate (NK cells) and adaptive cellular immune response (T cells). The cellular response eliminates infected hepatocytes by induction of apoptosis, elicited either by members of the TNF superfamily such as Fas ligand or TNF-related apoptosis-inducing ligand (TRAIL) or by granzyme B and perforin. However, activated effector CD8^+^ T cells also release TNF and other cytokines upon antigen recognition which facilitate viral control [70]. Although protective in *principle*, a sustained antiviral immune response mediates the immunopathogenesis of chronic viral hepatitis, as the viruses themselves are not cytopathic. With respect to HBV infection, polymorphisms in the TNF promoter region affect the susceptibility towards chronicity and disease pathology of hepatitis B (summarized in [71]).

Since HBV and HCV do not infect murine hepatocytes in vivo, several cellular studies analysed the role of viral proteins for sensitisation towards TNF-induced hepatocyte killing. For example, transfection of the HBV proteins HBx or HBV core protein (HBc) sensitized cell lines towards TNF-induced apoptosis [72, 73]. Besides its role for induction of cell death in HBV-infected hepatocytes, TNF directly controls HBV infection by blocking HBV replication through destabilisation of the viral nucleocapsid and decay of cccDNA [74], an effect that was reported to depend on NFκB activation [75] and c-Flip expression [76]. In an in vitro HCV infection model with Huh-7.5 cells, Park J et al. [77] demonstrated that HCV-infected cells were susceptible towards TNF-induced apoptosis compared to uninfected cells. Interestingly, HCV infection inhibited TNF-induced activation of IKK, nuclear translocation of NFκB, and expression of NFκB-dependent anti-apoptotic proteins, such as c-FLIP, XIAP, and Bcl-x_L_. mRNA transcripts of these anti-apoptotic proteins were also significantly reduced in liver tissue of patients with chronic hepatitis C compared to non-viral hepatitis. Transfection experiments revealed that TNF sensitisation of HCV-infected Huh-7.5 cells was probably mediated by the core, non-structural HCV proteins (NS)4B and NS5B [77].

Several studies analysed molecular and immunological mechanisms of TNF signalling during viral hepatitis in mouse infection models. TNF has been shown to promote hepatitis in murine hepatitis virus (MHV) infection as *Tnfr1*^*−/−*^ mice displayed less hepatic apoptosis and necrosis, and reduced neutrophil infiltration into the infected liver as well as prolonged survival [78]. The hepatoprotective effect of TNF in MHV infection seemed to be mediated by activation of NFκB in liver parenchymal cells, since conditional knockout mice that specifically lack RIPK1 in hepatocytes developed more severe hepatocellular damage and an aggravated inflammatory response. Hence, RIPK1 deficiency in hepatocytes mimicked the TNF response observed in presence of transcriptional inhibition by d-GalN [79].

In a recent study, Lampl and co-workers [80] provided evidence for a TNF-inducible, cell-autonomous mechanism that selectively eliminates virus-infected hepatocytes. This effect was observed in mice infected with a hepatotropic recombinant adenovirus and was mediated by TNFR1, but not by TNFR2, upon TNF challenge. Activation of immune cells by TNF or by the virus was not required. The cell-autonomous role of TNF was proven by administration of a Cre-recombinase encoding adenovirus that selectively deleted TNFR1 in infected hepatocytes of *Tnfr1*^*flox/flox*^ mice. In these animals, TNF challenge failed to induce liver damage. In TNFR1 expressing hepatocytes, TNF induced cleavage of caspases 8, 9, and 3. However, administration of the Cre-recombinase encoding adenovirus to *Casp8*^*flox/flox*^ mice failed to attenuate liver damage upon TNF injection, indicating that the observed mode of cell death was independent of caspase 8. Notably, the authors neither observed necroptosis, pyroptosis, ferroptosis, nor oxeiptosis upon TNF injection to adenovirus-infected mice. However, inhibitor studies and studies using genetically modified mice or calcium scavengers as well as determination of the mitochondrial membrane potential provided evidence for a reactive oxygen species (ROS) and calcium-dependent deterioration of the mitochondrial membrane potential of infected hepatocytes exposed to TNF in vivo, indicating that TNF-induced cell death in adenovirus-infected mice was characterized as a caspase 8-independent, MPT-dependent, non-canonical type of hepatocyte apoptosis.

Although TNF seems to control viral hepatitis by induction of cell death in virus-infected hepatocytes, TNF signalling might also favour viral persistence by impairment of the antiviral T cell response, either by induction of T cell apoptosis [81] or by enhancing T cell inactivation, also known as T cell exhaustion [82]. *Tnfr1*^*−/−*^ mice showed increased numbers of virus-specific memory CD8^+^ T cells upon infection with the hepatotropic lymphatic choriomeningitis virus (LCMV), compared to *Tnfr2*^*−/−*^ mice [81]. Interestingly, mice that lack both TNF receptors showed an even higher increase of LCMV-specific CD8^+^ T cell numbers. TNF receptor deficiency did not affect proliferation but rather reduced apoptosis of LCMV-specific CD8^+^ T cells, pointing to a role of TNF for T cell contraction during chronic viral infection. In another study, LCMV-specific T cells from chronically infected mice displayed increased expression of programmed cell death (PD)-1 and other co-inhibitory molecules, indicating enhanced T cell exhaustion [82]. Treatment of chronically infected mice with the anti-TNF antibody infliximab resulted in an increase of LCMV-specific CD4^+^ and CD8^+^ T cells, a concomitant reduction of PD-1 expression, and enhanced antigen-specific cytotoxicity of CD8^+^ T cells along with an improved control of viral infection. Interestingly, neutralization of TNF resulted in elevated levels of serum transaminases which was probably due to T cell–mediated hepatitis. The relevance of TNFR signalling for T cell immunity was analysed by adoptive transfer of CD4^+^ and CD8^+^ T cells from acutely infected wild-type or TNFR1/TNFR2 double knockout mice into chronically infected animals. Analysis of the transferred T cells demonstrated significantly increased frequencies of LCMV-specific TNFR-deficient T cells compared to wild-type T cells, decreased frequencies of PD-1^+^ T cells, and a concomitantly enhanced ability of TNFR-deficient T cells to clear the virus. Using transgenic mice, the authors demonstrated that continuous NFκB activation in LCMV-specific CD4^+^ T cells resulted in increased PD-1 expression and impaired helper function in infected mice, which resulted in substantially lower frequencies and reduced effector functions of LCMV-specific CD8^+^ T cells. Accordingly, the authors discovered NFκB responsive elements in the *PDCD1* locus. Together this study indicates that continuous NFκB activation in CD4^+^ T cells may provide a molecular link between TNF and T cell exhaustion during chronic viral infection. However, blockade of TNF during viral hepatitis might be contraindicated not only because of the well-documented side effects of reactivation of quiescent infections but also because of restricted elimination of infected hepatocytes.

In an immunocompetent mouse model of chronic HBV infection, using hydrodynamic injection of a HBV plasmid to wild-type and cIAP1 and 2–deficient mice, it was shown that cIAPs, which are activated in the TNFR-dependent NFκB pathway, attenuated TNF-induced cell death of HBV-infected hepatocytes, thereby allowing viral persistence [83]. Accordingly, inhibition of the function of IAPs by drug inhibitors of cIAPs, known as Smac mimetics, since they mimic the action of the endogenous protein Smac/Diablo that antagonises cIAP function, promoted the elimination of HBV [84]. In a similar mouse model of HBV-infected immunocompetent mice, TNF neutralisation by etanercept impaired viral clearance of HBV, associated with a higher number of intrahepatic PD-1^high^CD127^low^ exhausted CD8^+^ T cells [85]. Although there might exist virus-specific differences between LCMV and HBV infection regarding TNF-mediated exhaustion of helper and effector T cells, future studies are needed to elucidate the role of TNF and its receptors for T cell exhaustion during viral hepatitis.

## Role of TNF in autoimmune liver disease (AILD)

### Autoimmune hepatitis (AIH) and primary biliary cholangitis (PBC)

Despite the unknown aetiology of AILD, genome-wide association studies provided evidence for a strong association of AILD with human leucocyte antigen (HLA) loci, emphasizing a critical role of adaptive T cell responses in the immunopathogenesis of AILD [86, 87]. Preclinical studies using TNF neutralisation or *Tnfr1*^*−/−*^ and *Tnfr2*^*−/−*^ mice provided considerable evidence for a pathogenic role of TNF in acute T cell–dependent liver injury [27, 30, 40, 88, 89]. Pathway analysis in PBC patients provided evidence for the importance of TNF signalling [87]. Moreover, few clinical studies reported successful results of anti-TNF treatment in patients with RA and co-existing PBC [90, 91]. In patients with autoimmune hepatitis (AIH), increased frequencies of TNF-producing CD4^+^ T cells were detectable in peripheral blood and liver biopsy specimens compared to healthy controls. As the majority of TNF-producing CD4^+^ T cells also produced IFNγ, the authors concluded that these cells are pathogenically activated Th1 cells. Indeed, antigen-specific activation of T cells resulted in significant TNF production by CD4^+^ T cells from AIH patients who developed the corresponding antigen-specific antibody response [92]. In addition, a polymorphism in the TNF gene was associated with reduced remission, increased treatment failure, and enhanced incidence of liver cirrhosis in patients with-type 1 AIH compared to normal subjects [93]. Intriguingly, anti-TNF therapy (infliximab) of AIH patients not responding to standard treatment resulted in a significant reduction of serum transaminases and immunoglobulins [6]. Since anti-TNF treatment can be accompanied by infectious complications, the authors recommended infliximab as a rescue treatment for patients with severe and difficult-to-treat AIH. The mechanism of action of infliximab was discussed as neutralisation of soluble and transmembrane TNF and induction of apoptosis of activated lymphocytes. Indeed, besides inhibition of the production of other pro-inflammatory cytokines, anti-TNF antibodies induce apoptosis of mucosal T cells from Crohn’s disease patients, for example by direct activation of caspase-3 in activated CD4^+^ T cells, which is probably mediated by reverse signalling via mTNF, that can function as a receptor of the antibody (cf. Fig. 2) [94]. Hence, it could be speculated that a reduction of activated T cells has also been responsible for the effect of infliximab on AIH [6].

Overall, anti-TNF therapy can either ameliorate AIH or induce an immune-mediated liver disease resembling AIH [95], featuring periportal inflammation as well as elevated serum levels of liver enzymes and anti-nuclear antibodies in a large number of cases [96]. However, patients showed a good response to steroid therapy and failed to relapse after discontinuation of steroid treatment [96]. The paradoxical clinical effects of anti-TNF treatment are possibly due to multiple responses of CD4^+^ T cells to TNF. TNF, via TNFR1, pre-dominantly activates the innate immune response including maturation of dendritic cells, thereby priming CD4^+^ T cells [97]. On the other hand, TNF can induce apoptosis of highly activated effector T cells, thereby determining the pool size of pathogenic conventional T cells [98]. Intriguingly, activation of either TNFR1 or TNFR2 seems to be important for the response of CD4^+^ T cells to TNF. In contrast to the ubiquitous expression of TNFR1, TNFR2 expression is restricted to certain cell types including lymphocytes. It is well documented that TNFR2 is preferentially expressed by human and mouse CD4^+^Foxp3^+^ Tregs [14, 15]. TNFR2 stabilizes Foxp3 by preventing DNA methylation at the *Foxp3* promoter, and prevents pathogenic conversion of Tregs under inflammatory conditions [99]. Moreover, TNFR2 increases the suppressive function of Tregs, most likely by induction of their signature molecules CD25, CTLA-4, and GITR, as well as Treg survival via induction of Bcl-2, and Treg proliferation (cf. Fig. 2) [14]. In mouse arthritis models, it has been shown that *Tnfr1*^*−/−*^ mice were protected whereas *Tnfr2*^*−/−*^ mice displayed disease exacerbation. Accordingly, treatment with either TNFR1 antagonists or TNFR2 agonists ameliorated the disease (Table 2) [15]. Taken together, the disparate function of TNFR1 and TNFR2 in T cell immunity underscores the necessity to selectively analyse their cell-specific role in preclinical mouse models of immune-mediated liver injury and to develop novel therapeutic agents that selectively block TNFR1 and, on the contrary, selectively activate TNFR2 for treatment of autoimmune liver disease.

**Table 2:** Therapeutic effects of next-generation biologicals targeting TNFR1 or TNFR2 in models of autoimmune disease and liver disease ( adapted from 15 and 126)

Agent	Structure	Therapeutic efficacy
TNFR1 antagonists		
XPro1595	Mutated human sTNF unable to signal through TNFR1, XENP345: pegylated form	EAE, LPS-induced hepatitis, high-fat diet induced insulin resistance
R1antTNF	sTNF mutein, PEG-R1antTNF: pegylated form	ConA- and CCl_4_-induced hepatitis, EAE, CIA
Atrosab	Humanized anti-TNFR1 IgG1 mAb	EAE, CIA, NASH
TROS	Trivalent nanobody	EAE, acute huTNF-induced liver inflammation in uPA^+/+^-SCID mice
TNFR2 agonists		
STAR2	Mouse TNF mutein nanomer	Increased expansion, survival and function of T_regs_; CIA, EAE, GvHD
EHD2-sc-mTNF_R2_	Mouse TNF mutein hexamer	Increased expansion of T_regs_; CIA, EAE
MR2-1	Anti-human TNFR2 (mouse mAb)	Increased expansion and stability of T_regs_; not tested in vivo

*CCl*_*4*_, carbon tetrachloride; *CIA*, collagen-induced arthritis; *ConA*, concanavalin A; *EAE*, experimental autoimmune encephalomyelitis; *GvHD*, graft-versus-host disease; *LPS*, lipopolysaccharide; *mAb*, monoclonal antibody; *TNF*, tumour necrosis factor; *TNFR*, TNF receptor; *TROS*, TNF receptor one silencer

### Primary sclerosing cholangitis (PSC)

PSC is a chronic cholestatic liver disease characterized by biliary inflammation and progressive fibrosis, end-stage liver disease, and a high risk of malignancy. Current medical treatment of PSC is of limited effectiveness. More than 80% of PSC patients have IBD with a unique clinical phenotype [100]. Alterations in BA composition and reabsorption of toxic BAs seem to be involved in the pathogenesis of PSC [58], a mechanism which has been recently observed to be regulated by T cells and inflammatory cytokines in a mouse model [60]. Indeed, PSC has been recognized as an immune-mediated biliary disease. A large majority of PSC-associated gene loci involve molecules essential for immune-related pathways. Strong associations are described for HLA loci and genes of the interleukin (IL)-2 pathway (*CD28, IL-2*, and *IL-2RA*), suggesting that T cell–dependent adaptive immune responses contribute to the immunopathogenesis of PSC [101]. Recently, these genetic association studies have been reinforced by functional studies indicating failure of activated CD4^+^ T cell apoptosis [102] and reduced circulating frequencies of Tregs in PSC patients [103]. In addition, gene variation analysis indicated a significant reduction of HLA alleles specific for inhibitory NK cell receptors in patients with PSC [104]. Indeed, circulating and hepatic NK cells from PSC patients harbour a specific phenotype and are enriched in liver tissue [105, 106]. Moreover, we recently demonstrated that NK cell– and IFN-γ-dependent immune responses contribute to the pathogenesis of sclerosing cholangitis [105].

Emerging evidence from clinical and preclinical studies indicates the development gut dysbiosis in PSC patients [107] and in mouse models [108, 109]. In addition, these studies point to a role of an inappropriate immune response towards the altered commensal microbiota for PSC disease pathology. For example, an increased Th17 response to biliary pathogens has been observed in PSC patients [110]. Th17 cells are abundant in the intestinal lamina propria where they are induced by commensal bacteria and provide protection against invading pathogens. Pathogenic microbes may induce a dysregulated IL-17 response which is critically involved in IBD [111]. Recently, a single cell atlas of intrahepatic CD4^+^ T cells of PSC patients has been established, in which single cell sequencing and trajectory analysis provided evidence for expansion of tissue resident CD4^+^ T cells exhibiting a phenotype of naïve T cells that are prone to polarize towards effector Th17 cells [112]. Amongst the ‘non-classical’ innate-like T cells, resembling mucosal-activated invariant T cells, three clusters with distinct functional phenotypes were identified, amongst which are Th17 like innate cells, expressing the transcription factor *RORA*, as well as pro-inflammatory cells, characterized by expression of high levels of TNF [112]. Hence, Th17 cells expressing IL-17, IL-23, and other cytokines as well as T cells and innate lymphocytes expressing IFNγ and TNF have been associated with disease pathology of PSC. Several small cohort studies in PSC and PSC/IBD patients analysed the impact of anti-TNF therapies, which seemed to be only moderately effective [113, 114]. It was concluded that the heterogeneity of the phenotype in PSC possibly makes it difficult to draw clear conclusions from studies involving only a small number of patients [113].

Two well-accepted murine models serve as animal models for sclerosing cholangitis. These are (i) the *Mdr2*^*−/−*^ mouse model, bearing a targeted disruption of the multidrug resistance gene that corresponds to the human orthologue *MDR-3* [115] and (ii) the model of 3,5-diethoxycarbonyl-1,4-dihydrocollidine (DDC)-fed wild-type mice [109]. Liver histopathology in these models displays specific morphological features observed in livers of PSC patients, such as biliary inflammation, ductular proliferation, and onion skin type periductal fibrosis. Preclinical studies in these models showed dysbiosis and increased gut permeability in *Mdr2*^*−/−*^ mice and a good response to antibiotic treatment in DDC-fed animals [109]. Moreover, these mice mounted a hepatic IL-17 response which depended on intestinal pathobionts, epithelial barrier disruption, and bacterial translocation. IL-17 was produced by either γδT cells (in *Mdr2*^*−/−*^ mice [108]) or Th17 cells (in gnotobiotic DDC-fed mice recolonised with faecal samples from PSC/IBD patients [109]).

Enhanced levels of TNF have also been detected in *Mdr2*^*−/−*^ mice compared to wild-type mice [105]. A preclinical functional study in the DDC model indicated that anti-TNF treatment had no effect on serum levels of liver enzymes but prevented the ductular reaction as well as extracellular matrix deposition in mouse livers [116]. We wondered whether TNF would affect liver inflammation, liver damage, and fibrosis in chronic experimental sclerosing cholangitis in the *Mdr2*^*−/−*^ mouse model. Using *Mdr2*^*−*/*−*^ × *Tnfr1*^*−/−*^ double knockout mice, we observed an aggravated disease pathology of sclerosing cholangitis associated with an increased Th17 response in *Mdr2*^*−*/*−*^ × *Tnfr1*^*−/−*^ mice compared to *Mdr2*^*−/−*^ mice [117]. Moreover, Th17-related chemokine expression (*Ccr6* and *Cxcr6*) and expression of *Ccl2* and *Cx3cl1*, which are critically involved in the recruitment of inflammatory monocytes, were also elevated. Accordingly, we observed increased frequencies of CD11b^+^CX_3_CR1^+^ monocytes/macrophages in livers of *Mdr2*^*−*/*−*^ × *Tnfr1*^*−/−*^ mice compared to *Mdr2*^*−/−*^ mice which changed their phenotype by inappropriately expressing *Ripk3*. CX_3_CR1^+^ RIPK3^+^ macrophages have been recently shown to produce the cytokines IL-1β and IL-23 which promote Th17 responses [118].

Since *Mdr2*^*−/−*^ mice are a mouse model of inflammation-induced tumour development, and IL-17 has been closely associated with strong induction of regeneration and angiogenesis in the tumour microenvironment [119], we analysed gene expression of cell proliferation markers and known tumour markers of HCC in livers of *Mdr2*^*−*/*−*^ × *Tnfr1*^*−/−*^ compared to control mice. We observed a significant increase of the expression of *Pcna* (gene of proliferating cell nuclear antigen), *Ccna2* (gene of cyclin A2), Cdk1 (gene of cyclin-dependent kinase 1), *Ssp1* (gene of secreted phosphoprotein-1, OPN), and *Afp* (gene of α-feto protein) in the double knockout mice, indicating that they might be more susceptible to development of HCC than *Mdr2*^*−/−*^ mice. As mentioned earlier, activation of TNFR1 induces cell survival and liver regeneration by activation of NFκB. Interestingly, targeted disruption of NFκB signalling specifically in intestinal epithelial cells in the gut resulted in intestinal barrier disruption and chronic colitis, which did not develop under germ-free conditions [120]. Moreover, hepatocyte-specific deletion of IKKβ, a subunit of the kinase complex that activates NFκB (cf. Fig. 1), severely aggravated liver damage and fibrosis in *Mdr2*^*−/−*^ mice [121]. Therefore, with respect to our study, it seems that TNFR1 might have been responsible for NFκB activation in hepatocytes and in intestinal epithelial cells thereby supporting cell survival and preventing intestinal dysbiosis, intestinal barrier dysfunction, and an inappropriate immune response which aggravates sclerosing cholangitis in a susceptible host.

## Role of TNF in hepatocellular carcinoma (HCC)

Functional evidence for a role of TNF in HCC was provided by studies in mice. Deficiency of the TNFR-dependent anti-apoptotic NFκB signalling pathway was shown to be essential for induction of compensatory proliferation of live hepatocytes in response to hepatocyte death which results in development of HCC [7]. Mice lacking the regulatory subunit of the IKK complex, NEMO, specifically in hepatocytes, spontaneously develop chronic liver disease that progresses to HCC [122] due to increased TNFR1-dependent death of NEMO-deficient hepatocytes [123]. Moreover, hepatocyte-specific deletion of IKKβ exacerbated chemically induced liver cancer in mice, probably by aggravating carcinogen-induced hepatocyte death and induction of compensatory proliferation of hepatocytes [124]. However, inhibition of NFκB activation by anti-TNF treatment or induction of a (transgenic) non-degradable form of IκB at late stages of tumour development in *Mdr2*^*−/−*^ mice resulted in apoptosis of transformed hepatocytes and lack of progression to HCC [125]. Hence, early TNFR1-inducible NFκB activation associated with reduced hepatocellular death seems to be linked to an inhibition of compensatory hepatocyte proliferation in *Mdr2*^*−/−*^ mice, indicated by a reduced expression of proliferation and tumour markers [117], while at later time points NFκB mediates survival of transformed hepatocytes that is responsible for the progression to HCC [125].

## Conclusion and therapeutic outlook

By activation of the ubiquitously expressed TNFR1, TNF provides signals for eradication of pathogens and tissue damage such as inflammation and cell death, but also induces cell survival, proliferation, and tissue regeneration, thereby contributing to resolution of inflammation and tissue repair. Therefore, it is not surprising that anti-TNF therapy has major beneficial clinical effects in several autoimmune diseases such as RA and IBD, but also induces adverse effects such as increased susceptibility to infection and more unexpectedly exacerbation of pre-existing autoimmune disease (e.g. multiple sclerosis) or even new development of autoimmune-like symptoms in different organs [2, 8]. In the liver, evidence for therapeutic effects of anti-TNF treatment is accumulating, while induction of DILI, autoimmune-like hepatitis, and cholestatic hepatitis have been reported in rare cases that are, however, often reversible after anti-TNF withdrawal [95].

While the role of TNFR1 for liver inflammation, induction of several forms of hepatocyte death, cell survival, liver regeneration, hepatocyte proliferation, and tumorigenesis is well documented, the role of TNFR2 for acute and chronic liver injury is scarcely described. A few reports suppose that TNFR2, although devoid of a death domain and primarily activating NFκB, might facilitate TNFR1-induced liver cell death [27, 40], a mechanism which has been described to depend on delocalisation and degradation of TRAF2 and negative regulation of NFκB signalling (reviewed in [2]). However, in view of the immuno-regulatory nature of TNFR2, which plays an important function for Treg stability, immunosuppressive function, and expansion [14, 15], preclinical studies in chronic models of liver injury using TNFR2-deficient or, even more important, cell-specific TNFR2 knockout mice are urgently needed. With respect to therapy of inflammatory and autoimmune liver disease, future trials should investigate the therapeutic efficacy of selective TNFR1 antagonists that inhibit liver inflammation while sparing the suppressive functions of Tregs. On the other hand, activation of TNFR2 by agonistic antibodies or agonistic TNF muteins that activate, stabilize, and expand Tregs [15, 126] could have considerable therapeutic implications for treatment of autoimmune liver disease. A list of next-generation biologicals specifically targeting TNFR1 or TNFR2 in models of autoimmune diseases and liver disease is given in Table 2. In contrast to these considerations, TNFR2 antagonism could be relevant for treatment of cancer, considering that TNFR2^+^ Tregs could suppress the antigen-specific T cell response against the tumour [127]. Taken together, with respect to successful therapy of inflammatory and autoimmune diseases targeting TNF, a future approach will be selective inhibition or activation of one of the two TNFRs.
